# Social Adversity Impacts on the Rhesus Macaque Immune System Resemble Those of Aging

**DOI:** 10.1093/geroni/igaa057.3394

**Published:** 2020-12-16

**Authors:** Mitchell Sanchez-Rosado, Noah Snyder-Mackler, Lauren Brent, James Higham, Clare Kimock, Marina Watowich, Melissa Pavez-Fox, Petraleigh Pantoja-Maldonado

**Affiliations:** 1 University of Puerto Rico Medical Sciences Campus, San Juan, Puerto Rico; 2 Arizona State University, Tempe, Arizona, United States; 3 University of Exeter, Exeter, United Kingdom; 4 New York University, New York, New York, United States; 5 University of Washington, Seattle, Washington, United States

## Abstract

Social adversity can impact immune function and is associated with increased morbidity and mortality. Many of these immune-related changes resemble the effects of the natural aging process. To date, little is known about the effects of social adversity on the immune system change across the lifetime. Here, we investigated how aging and social adversity (measured as social status) impact immune cell proportions. We performed flow cytometry on peripheral whole blood from a population of free-ranging rhesus macaques to quantify changes on immune cell proportions across the lifespan (n=99) and across different social statuses (n=53). Overall, we found that high adversity recapitulated the effects of aging. We found significant shared decreases in CD3+/CD4+ T cell proportions and corresponding increases in CD3+/CD8+ T cell proportions between individuals of older ages and low social status. These findings suggest that social adversity has similar effects to aging on T cell proportions, possibly affecting the T cell component of the immune response. In contrast, CD3+/CD4+/CD25+ T regulatory cell proportions increased with age, which is typical of normal aging. Contrary to our expectations, these cells were less abundant in low status individuals, indicating some overall regulatory immune deficits specific to lower status individuals. CD3+/CD8+/CD25+ T regulatory cells, which contribute to self-tolerance, were higher in high status individuals, suggesting that overall primary immune regulatory cells can be affected by social adversity and impair the regulation of inflammation. We provide evidence that social adversity alters immune cell proportions, implicating it in the development of inflammatory and/or aging-related diseases.

